# Precision and reliability study of hospital infusion pumps: a systematic review

**DOI:** 10.1186/s12938-023-01088-w

**Published:** 2023-03-17

**Authors:** Mayla dos S. Silva, Joabe Lima Araújo, Gustavo A. M. de A. Nunes, Mário Fabrício F. Rosa, Glécia V. da Silva Luz, Suélia de S. R. F. Rosa, Antônio Piratelli-Filho

**Affiliations:** 1 Postgraduate Program in Mechatronic Systems, at Mechanical Engineering Department, University of Brasília, Brasília, DF Brazil; 2 Postgraduate Program in Biomedical Engineering, at Faculty of Gama-FGA. University of Brasília, Brasília, DF Brazil; 3 Institute of Biology, Department of Genetics and Morphology, University of Brasília, Brasília, DF 70910–900 Brazil; 4 Department of Collective Health, Faculty of Ceilândia. University of Brasilia, Brasília, DF, Brazil

**Keywords:** Infusion pumps, Precision, Reliability, Medical equipment

## Abstract

**Background:**

Infusion Pumps (IP) are medical devices that were developed in the 1960s and generate fluid flow at pressures higher than that of normal blood pressure. Various hospital sectors make use of them, and they have become indispensable in therapies requiring continuity and precision in the administration of medication and/or food. As they are classified Class III (high risk) equipment, their maintenance is crucial for proper performance of the device, as well as patient and operator safety. The principal consideration of the pump is the volume infused, and the device demands great attention to detail when being calibrated. A lack of necessary care with this equipment can lead to uncertainty in volume and precision during the administration of substances. Because of this, it is essential to evaluate its reliability, to prevent possible failures at time of execution. This control aims at the quality of the intended infusion result, becoming an indication of quality.

**Methods:**

This systematic review summarizes studies done over the last 10 years (2011 to December 2021) that address the reliability and accuracy of hospital infusion pumps, in order to identify planning of maintenance and/or other techniques used in management of the equipment. The Prisma method was applied and the databases utilized were Embase, MEDLINE/Pubmed, Web of Science, Scopus, IEEE Xplore, and Science Direct. In addition, similar reviews were studied in Prospero and the Cochrane Library. For data analysis, softwares such as Mendeley, Excel, RStudio, and VOSviewer were used, and Robvis helped in plotting risk of bias results for studies performed with Cochrane tools.

**Results:**

The six databases selected produced 824 studies. After applying eligibility criteria (inclusion and exclusion), removing duplicates, and applying filters 1 and 2, 15 studies were included in the present review. It was found that the most relevant sources came from the Institute of Electrical and Electronics Engineers (IEEE) and that the most relevant keywords revolved around the terms (“device failure”, “infusion pumps”, “adverse effects”, “complications”, etc.). These results made clear that there remains substantial room for improvement as it relates to the study of accuracy and reliability of infusion.

**Conclusions:**

We verified that the reliability and precision analysis of hospital infusion pumps need to be performed in a more detailed and consistent way. New developments, considering the model and IP specification, are intended, clearly explaining the adopted methodology.

**Supplementary Information:**

The online version contains supplementary material available at 10.1186/s12938-023-01088-w.

## Background

The first uses of intravenous therapy were recorded in the 1600 s, and were done with experiments using animal feathers, and bladders as infusion materials [1]. Following the Second World War, this type of therapy was introduced as a routine practice in healthcare [1]. The first automatic equipment, built by the German scientist Watkins, had electromechanical characteristics; and was called the “chronofuser” [2]. At that time, knowledge of micro-controlled devices and digital electronics was almost non-existent [2]. A variety of medical treatments are performed through infusion, including use of sedatives, analgesics, hormones, parenteral nutrition, and chemotherapeutic agents, among others. Infusions may have varying degrees of duration, depending on their specific use, and require great safety and precision [3]. With the modernization of health systems, Infusion Pump (IP) equipment was developed and implemented in healthcare centers in the 1960 s [4]. It is a device capable of generating the flow of a given fluid, at pressures that are higher than that of regular blood pressure for the intended location [5].

We found a small number of published studies describing the evaluation of reliability in infusion pumps. In view of this, more research related to the types of procedures performed and their related maintenance programs is necessary to ensure stable operation. These technological analyses are necessary to prevent sudden interruptions, as well as to minimize worst case scenarios, such as complete machine shutdown. As such it is essential to verify which types of testing and evaluation procedures have been conducted to keep them working properly.

To the best of our knowledge, a systematic review on the subject has not yet been published. Therefore, considering the importance of studies on equipment reliability (errors related to installation, maintenance, failures, and operating conditions), the objective of this review is to summarize published data on dependability and accuracy in infusion pumps. We investigated whether these and other variables have been evaluated and/or quantified over the last 10 years, as well as looking at current evidence for potential strategies to minimize risk of failure for this critical medical equipment.

### Infusion pumps

**Fig. 1:**
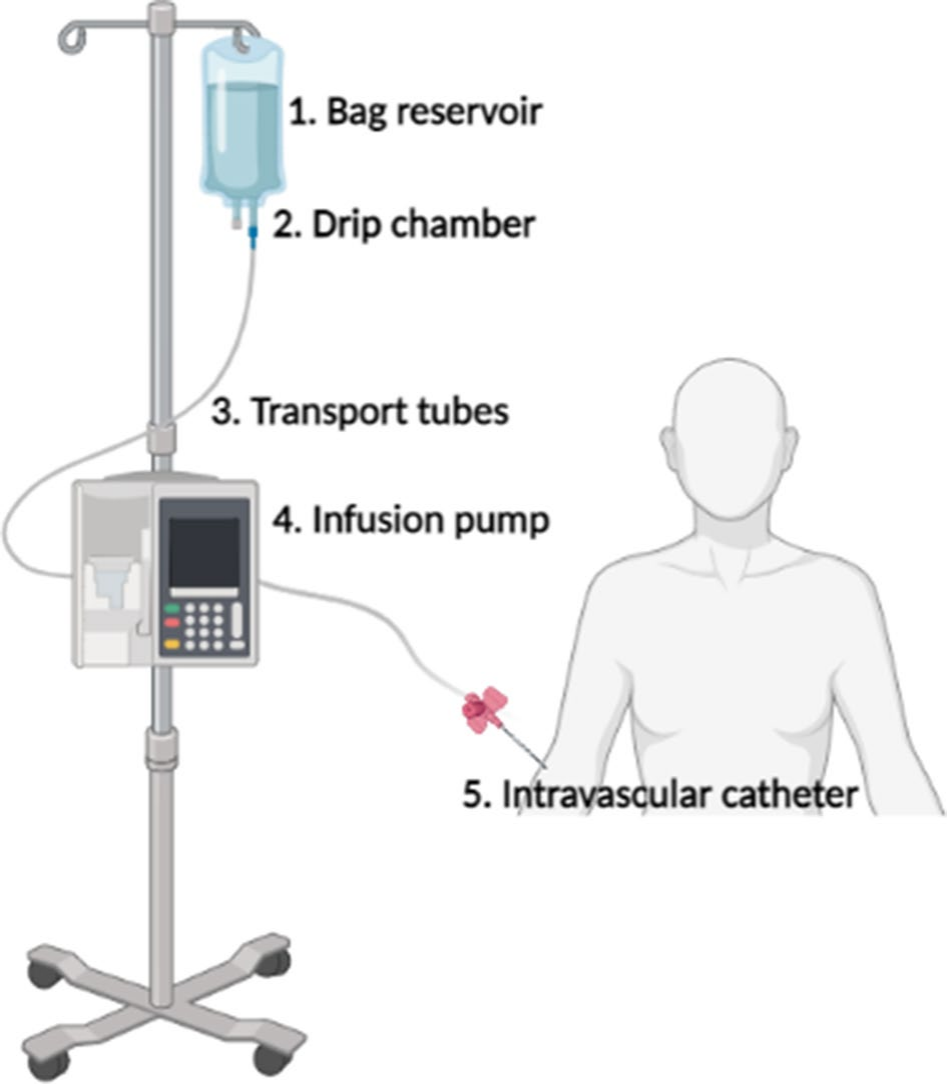
Representation of an infusion system including the infusion pump

The use of IPs, because of their specific alarms and controls, allows for accurate and safe infusion of liquids into the body [4]. The literature states that they require positive pressure to function which requires a directing mechanism. These operating principles can be peristaltic (rotary or linear), syringe or piston driven [6]. A modern infusion system consists of a percutaneous instrument (intravascular catheter) that passes through the skin to infuse fluids into a vein, a tube for transporting the liquid or a reservoir, containing the fluid to be delivered, which can be a bag or syringe, and a flow control device capable of stopping or regulating the infusion [3]. This system is shown in Fig. 1, including the IP.

As it is a mechanism directly connected to the patient, the attention to detail and care used with this equipment is critical. Intravenous infusions are the most common type of infusion and if they fail can cause aggravating problems for the patient and/or worsen their health condition, for example, causing venous spasm, pulmonary edema, and phlebitis [7, 8].

In the hospital environment, infusion pumps are most commonly found in Intensive Care Units (ICUs), Neonatal Intensive Care Units (NICU), and Surgical and Oncology Centers [9]. The process performed by infusion pumps is the automation of a repetitive hospital technique and is of great relevance to healthcare professionals, and directly impacts patients’ lives. Despite their importance, they are medical devices associated with the highest number of technical complaints, or adverse events, according to the techno-surveillance unit of the Agência Nacional de Vigilância Sanitária (ANVISA) [10]. This device is one of the most used Medical Assistance Equipment’s in ICU’s and is responsible for 19.4% of all adverse effects originated in the general context of the hospital environment, resulting from failures in drug administration [11].

The COVID-19 pandemic led to a dramatic increase in the use of ventilators. This increase, and the accompanying discomfort experienced by patients on breathing machines resulted in greater demand for the delivery of sedative infusions with bags requiring changing every 2–3 h [12]. Faced with this challenge, in April 2020 the Food and Drug Administration published changes related to the handling of pumps. The new guidelines particularly involved the ability to remotely monitor and adjust pump parameters throughout this public health emergency [12].

These decisions increased availability of equipment and helped reduce exposure of healthcare professionals to patients afflicted by the disease [13]. Other studies addressed the use of the devices outside patient beds and analyzed the handling and safety of the user [13–15]. However, despite this commitment to minimizing impacts of the pandemic, Nova Scotia Health’s leadership noted that a shortfall of infusion pump availability still persists in hospital settings [16].

### Reliability in medical equipment

Reliability of medical instruments can be understood as the probability of performing a given task without failure during a specific time interval under stated conditions and it is increasingly important for patient security. New developments in this area permit the use of new instrument models for in-home treatment of illnesses, as well as those for traditional hospitals and clinics settings. Hence, reduction and prevention of operating failures is critical and can be achieved by applying reliable regulation, rigorous maintenance and a warranty control plan [17].

Governments play an important role in the dependability of medical products. In countries like the United States and the European Union, regulations are established to guide direction of design and development phases of medical instruments, where manufacturers must anticipate and address any and all deficiencies that could lead to potential failure. In this respect, there are three distinct classes of instruments that require different approaches: general controls (class I), general and special controls (class II) and general controls and market approval (class III). According to Hedge (2008), infusion pumps are heavily regulated items and require specific labeling, adherence to performance standards and product tracking. As a result, the Medical industry must have solid programs in place that address concept, design, prototype and manufacturing phases as part of any new product development. Products already in use can be assessed using dependability tests, performed on units to induce failure modes, or to anticipate potential failures and implement corrective actions [17]. The author highlights known reliability tools that are recommended at each step, but claims that they are just starting to be implemented [17, 18].

Infusion pumps are instruments largely used in hospitals and their consistent, quality operation must be guaranteed. Failures in operation are generally registered in databases, together with maintenance data and classified as failure modes, operation times, repair times, and actions performed. In general, manufacturers are called to make repairs when necessary. However, due to the sheer number of infusion pumps in use at any given time in the hospitals reliability tests, and correlated data science tools could be applied to extract valuable information. The regular and consistent use of these types of equipment requires the utmost care to ensure safety of patients and operators. It is recommended that multiple studies on the reliability of medical equipment should be carried out around the world, to reduce possible errors in diagnoses, tragedies, injuries, economic losses, and other possible damage [18, 19]. Our proposal begins to close gaps in existing research.

## Methods

The present systematic review was conducted to summarize the last 10 years of research on the reliability and accuracy of hospital infusion pumps. The identification of effective maintenance planning and other equipment management techniques is the goal of this research.

### Protocol and registration

The present study was conducted according to PRISMA (Preferred Reporting Items for Systematic Reviews and Meta-Analyses) guidelines [20]. The protocol for this study was registered with the International Prospective Register of Systematic Reviews (PROSPERO) [21] under registration number: CRD42022304368. We used reference management software Mendeley^®^ and classified the folders according to the databases consulted. We used the RStudio (Bibliometrix library), and VOSviewer 1.6.8. software.

### Eligibility criteria

#### Inclusion Criteria

This review established inclusion criteria using the PICO approach (Population, Interest, Comparison, Outcome) [21]. (P) Research problem/Patient, population: hospital infusion pumps; (I) Interest: trials, tests, calibration; (C); not applicable; (O) Primary outcome: reliability study. Secondary outcome: main causes of pump failures, and precision rates. Filters were applied to limit the period (2011–2021), and to select conference (IEEE) and journal articles as well as language (English or Portuguese).

#### Exclusion Criteria

The following types of studies were excluded: (i) reviews, letters, personal opinions, book chapters and conference abstracts; (ii) articles that did not meet the inclusion criteria; (iii) studies of other types of equipment, such as implantable pumps; (iv) publication in languages other than English or Portuguese; (v) studies where the full paper copy was not available; (vi) low quality studies and (vii) studies done outside of the selected period (2011–2021).

### Information sources and search strategy

The creation of one single type of search strategy is generally not possible. Therefore, we elaborated on different combinations using Boolean operators, Individual search strategies for each of the following bibliographic databases were looked at: Embase, MEDLINE/PubMed, Web of Science, Scopus, IEEE Xplore, and Science Direct and the strings adapted for each database are listed in Table 1. In addition to the 6 databases, we verified the existence of similar studies in the Prospero and Cochrane Library, where we did not obtain results. The database search was conducted on October 10, 2021, with no time restriction. Duplicate references were removed using Reference Manager Software (Mendeley^®^). Articles were organized and analyzed systematically as the review provides an emerging observance in the COVID-19 pandemic, a fact that intensified the use of this equipment. To produce literature that was cohesive and integrated properly, we defined time limitations as contributions from January 2011 to December 2021, a period recognized for significant updates in the area of medical equipment enhanced standard adoption, for example, NBR IEC 60601–2-24.

**Table 1:** Database search strings

Data base	String
MEDLINE/PubMed	((((((“Infusion Pumps”[Mesh])) OR “infusors”[Mesh]) OR “Drug Infusion System”[Mesh]) OR “Perfusion Pumps”[Mesh]) AND ( “Equipment Failure”[Mesh] OR “Equipment Failure Analysis”[Mesh])) AND “adverse effects” [Subheading] AND (y_10[Filter])
Embase	((’infusion pump’/exp OR ’infusion’/exp) AND ’device failure’/exp OR ’device failure analysis’/exp) AND ’error’/exp
IEEE Xplore	infusion pump OR infusion pumps OR infusors OR Drug Infusion System OR Perfusion Pumps AND device failure OR device failure analysis AND accuracy AND maintenance
Scopus	( TITLE-ABS-KEY ( infusion AND pumps ) OR TITLE-ABS-KEY ( perfusion AND pumps ) OR TITLE-ABS-KEY ( infusors ) AND TITLE-ABS-KEY ( device AND failure ) OR TITLE-ABS-KEY ( device AND failure AND analysis ) AND TITLE-ABS-KEY ( error ) )
Web of science	“infusion pump” (Título) or “infusors” (Título) or “Perfusion Pumps” (Título) and “device failure” (Título) or “device failure analysis” (Título) or “error” (Título) and “accuracy” (Título) and “maintenance” (Título) and “adverse effects” (Título) and “reliability”
Science direct	(“infusion pump” OR “Perfusion Pumps” OR infusors OR “Drug Infusion System”) AND (“Equipment failure” OR “Equipment Failure Analysis” OR error OR “device failure” OR “device failure analysis”)

### Study and selection

The articles were selected in two phases: reading of titles and abstracts (phase 1) and reading of full text (phase 2). In phase 1, six authors (Silva, M. S.; Araújo, J. L.; Nunes, G. M.; Rosa, S. S. R. F.; Rosa, M. F. F.; Piratelli-Filho, A.) in blind pairs reviewed titles and abstracts of all references identified by the electronic databases which appeared to meet inclusion criteria and that respected eligibility criteria.

During phase 2, six pairs of authors (Silva, M. S.; Araújo, J. L.; Nunes, G. M.; Rosa, S. S. R. F.; Rosa, M. F. F.; Piratelli-Filho, A.) independently performed full text reviews of the articles, applying exclusion criteria. A third author was consulted in cases of disagreement (Luz, G. V. S.). Finally, the diagramming and extraction of the data obtained was organized in 3 written by the authors of the study and following verification protocol of PRISMA 2009 [20]. The excluded studies and the reason for exclusion are presented in the Additional file 1.

### Analysis of the findings

After the search and selection of studies of interest, the extracted data was tabulated using Excel spreadsheets for the development of analyses. To archive the bibliographic data, we utilized Mendeley software, from which we exported information to be evaluated with the help of the RStudio software (Bibliometrix library) and also VOSviewer 1.6.8. These tools make it possible to explore data such as scientific journals, identifying those with the greatest impact, the most influential authors, most common and relevant keywords, and other analyses.

### Risks of bias and quality in individual studies

Due to the heterogeneity of the techniques presented, we used narrative description with associated information, classification of the quality of studies, and minimization of bias through the Cochrane Collaboration Tool for Bias Risk Assessment applying a “Generic” platform. The platform can be adapted and has 7 domains, namely: (1) random sequence generation, (2)allocation concealment, (3) blinding of participants and personnel, (4) blinding of outcome assessment, (5) incomplete outcome data, (6) selective reporting and (7) other sources of bias. Hence, we evaluated the quality of the studies, where 4 independent reviewers (Silva, M. S., Rosa, S. S. R. F., Nunes, G. A., Piratelli-Filho, A.) analyzed risk of bias applying the methods described above. To visualize risks, we plotted figures using the Robvis tool, which is a web application aiding in the visualization of risk-of-bias [22]. Articles were classified as “high”, “low”, “no information” and “unclear” in regards to the risk of bias and “none” for missing data.

## Results

### Selection of studies

**Fig. 2:**
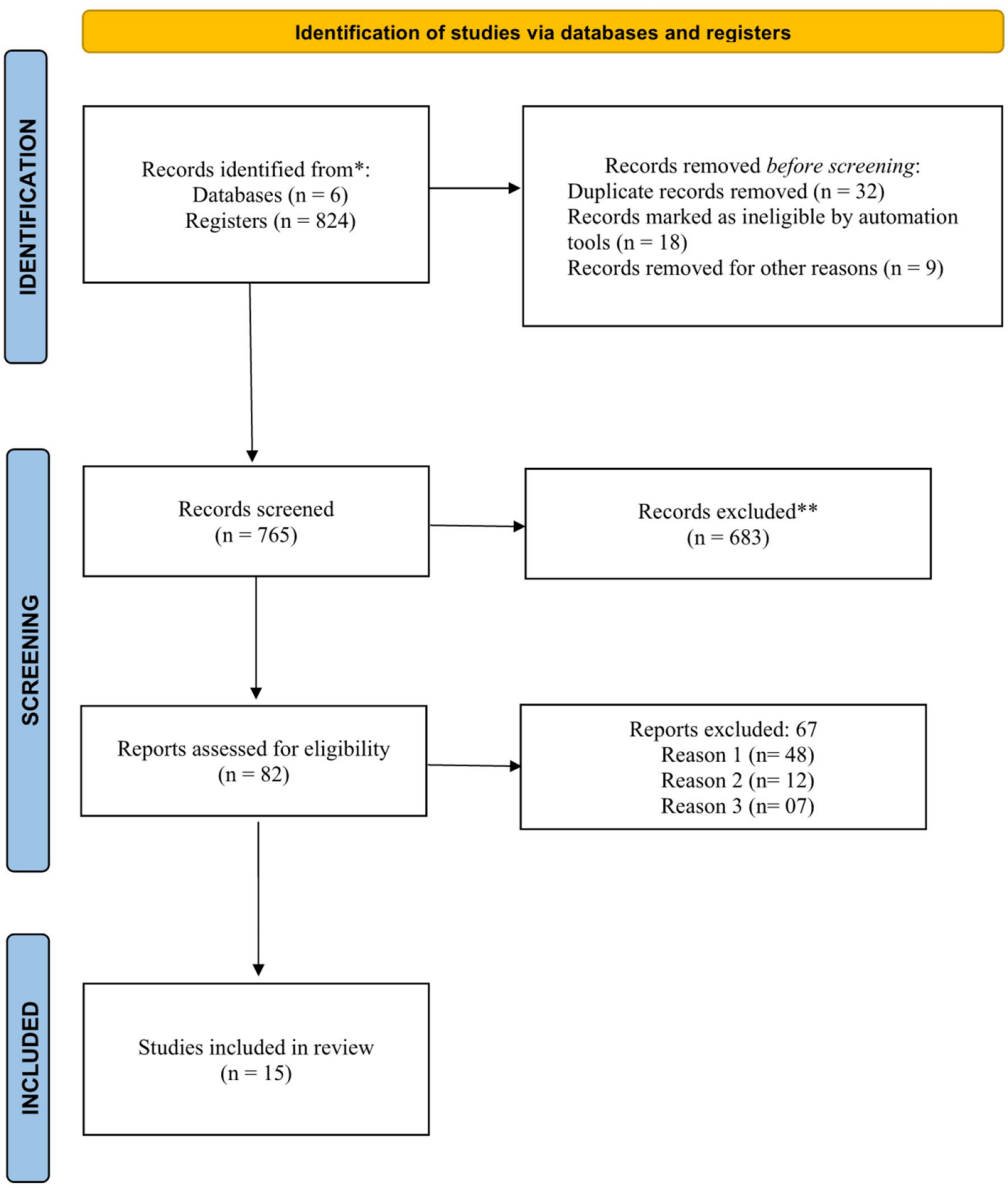
PRISMA 2020 diagram: methods and filters adopted in bibliographic-searches and selection criteria according to PRISMA 2020 protocol [20]

The search process for studies in 6 databases resulted in 824 studies. We applied criteria of inclusion, exclusion, and removal of duplicates. After employing these parameters we obtained the Prism diagram shown in Fig. 2.

### Process of analysis of the findings

After removing duplicates, 765 studies remained to be analyzed according to the first filter, that is, titles and abstracts. The main journals where these articles were published are listed in Table 2, which records prevalence of the engineering area. We note that the most relevant sources on the intended topic are predominantly found in the databases of the Institute of Electrical and Electronics Engineers (IEEE). For searches in the IEEE, we considered conferences and other related events, as they generally have high quality standards for acceptance. Of the 15 sources listed in Table 2, 8 are from the IEEE bases, the others are varied and from different health areas.

**Table 2:** Main journals that concentrate articles on the topic

Sources	Articles
IEEE TRANSACTIONS ON BIOMEDICAL ENGINEERING	30
BLOOD	17
JOURNAL OF DIABETES SCIENCE AND TECHNOLOGY	14
ANNALS OF SURGICAL ONCOLOGY	8
HEALTH DEVICES	8
IEEE ACCESS	8
2020 42ND ANNUAL INTERNATIONAL CONFERENCE OF THE IEEE ENGINEERING IN MEDICINE & BIOLOGY SOCIETY (EMBC)	7
IEEE JOURNAL OF BIOMEDICAL AND HEALTH INFORMATICS	7
ANESTHESIA AND ANALGESIA	6
IEEE TRANSACTIONS ON CONTROL SYSTEMS TECHNOLOGY	6
JOURNAL OF DAIRY SCIENCE	6
2011 ANNUAL INTERNATIONAL CONFERENCE OF THE IEEE ENGINEERING IN MEDICINE AND BIOLOGY SOCIETY	5
2014 36TH ANNUAL INTERNATIONAL CONFERENCE OF THE IEEE ENGINEERING IN MEDICINE AND BIOLOGY SOCIETY	5
2019 41ST ANNUAL INTERNATIONAL CONFERENCE OF THE IEEE ENGINEERING IN MEDICINE AND BIOLOGY SOCIETY (EMBC)	5
DIABETES TECHNOLOGY & THERAPEUTICS	5

**Fig. 3:**
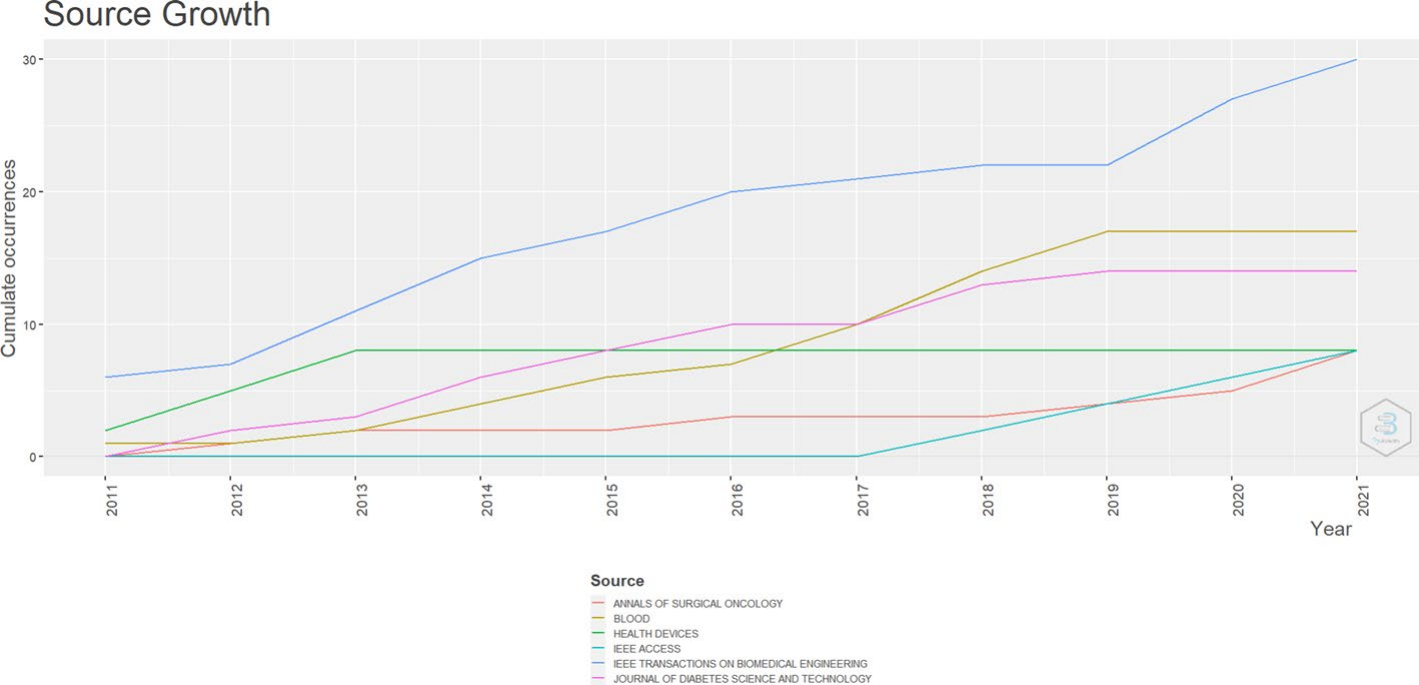
Representation of publications in the area from 2011 to 2021

The IEEE databases, in addition to leading in the number of studies in the area, have growth in publications from 2011 to 2021, as shown in Fig. 3. The journal IEEE Transactions on Biomedical Engineering presents 06 studies (year 2011), 07 studies (year 2012), 11 studies (in 2013), 15 studies (in 2014), 17 studies (year 2015), 20 studies (2016), 21 studies (2017), 22 studies (years 2018 and 2019), 27 (in 2020) and 30 publications on the subject in the last year (2021), as shown in blue in the graph. Then, we can see the Journal Blood, which remained constant from 2019 to 2021, with 17 studies each year, and the Journal of Diabetes Science and Technology with the same frequency of publications as the previous one (2019–2021), but with 14 studies per year.

**Fig. 4:**
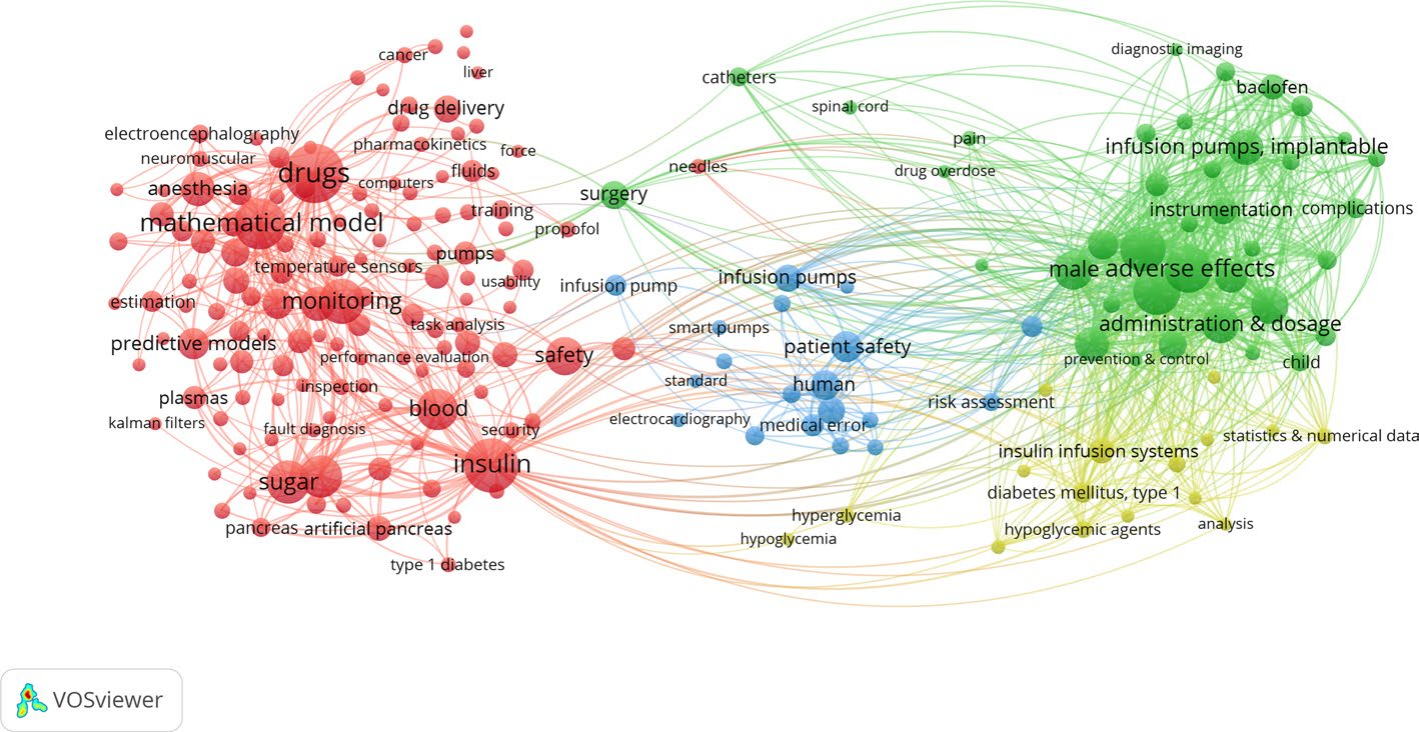
Bibliometric analysis of the 765 works obtained by searches in the data base. For this, the VOSviewer program version 1.6.1 was used, with the configuration: “full counting”, with at least 5x of terms co-occurrence

Using VOSviewer software, we analyzed the keyword network of search findings, as shown in Fig. 4. The nodes and their respective sizes represent the number of times, proportionally, that each word was cited. These nodes are connected if the words are co-quoted, in other words, cited by the same article. The connection between two nodes intensified depending on the number of co-citations. As a parameter, the software was informed that, to be considered a co-citation each word had to be mentioned at least 5 times, resulting in 205 nodes. This analysis was performed before the inclusion phase of the studies, allowing us to observe the strength of the words that involve insulin pumps and implantable ones, such as “insulin”, “sugar” and other terms. For the inclusion phase, we did not consider insulin pumps, so the area of interest of this review revolved around terms predominantly in highlighted in blue (“device failure”, “infusion pumps”, etc.) and green (“adverse effects”, “ complications”, etc).

**Fig. 5:**
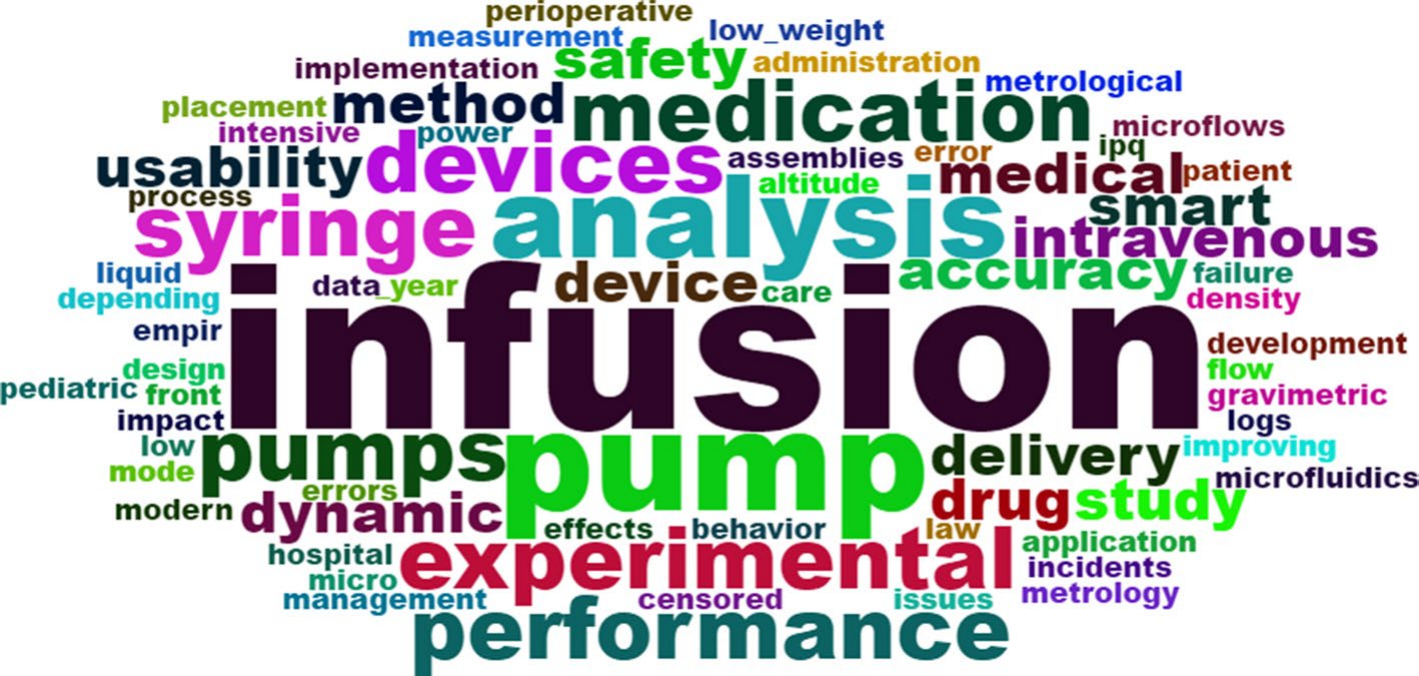
Word cloud produced from the words that appear most in the titles

Following the analyses presented above, with all the search findings (*n*= 765), we considered studies included after filters 1 and 2 were applied (*n*= 15). Using RStudio software, we adopted the Bibliometrix library to evaluate titles of the selected studies and plotted the word cloud shown in Fig. 5. The cloud represents the 70 most frequently used words in titles, with the term “pump” appearing most often (8 times) among the 15 titles.

**Fig. 6:**
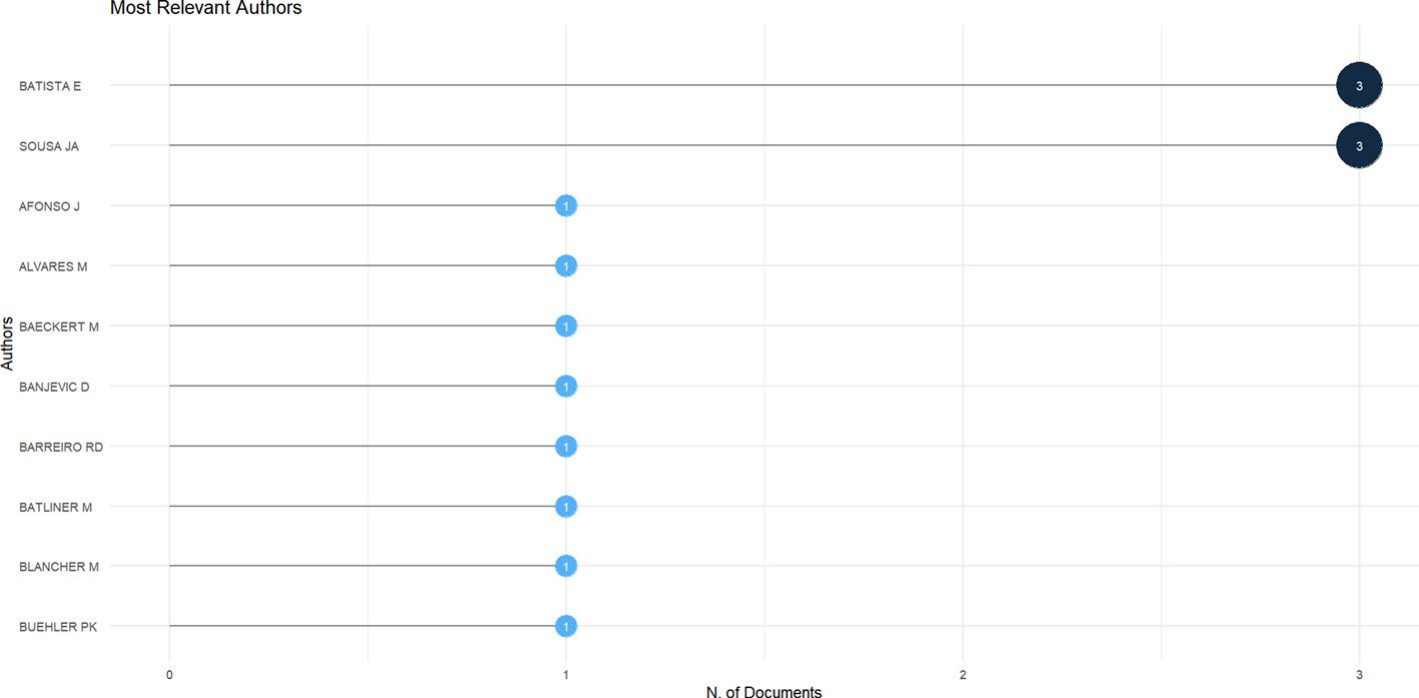
Most relevant authors among the inserted studies

From the included studies, 3 list Elsa Batista as the primary author [23–25], highlighting her as being influential in this area of studies, as shown in Fig. 6. João A. Sousa is the second most cited author in the sample.

The included studies and their main characteristics are presented in Table 3. The 15 studies included were developed in different countries with 20% of the studies done in Portugal, 13% in Brazil, 13% in the United States, and 7%, respectively, for Canada, France, Hong Kong, Iran, Korea, London, Spain and Switzerland.

**Table 3:** Characteristics of studies included in the review

Study	Author, year, local	Sample/ pump types	Intervention/ tests	Outcomes
[26]	N. Samaranayake et al., 2012, Hong Kong	1538 incident reports involving IP were randomly selected and evaluated. The pumps were of varying models and makes that were not specified.	Technology-related errors were analyzed to identify the type of technology responsible and the causes. Errors were divided into (i) Technology-related errors; (ii) Errors not related to technology. Within group (i) there was a division into: sociotechnical errors (which have human interaction) and device errors (related to technical defects of the device).	Of the errors involving technologies, 75.3% were related to prescription, 14.8% to medication administration, 8.4% to dispensing and 2.7% to other causes. Infusion pumps were responsible for 5.8% of sociotechnical errors. Device errors have only been observed with the use of infusion pumps.
[27]	A. Golpaygani et al., 2017, Iran	50 infusion pumps from 04 different brands and models were not specified.	Tests conducted to verify that the device pumps the required flow rate, volume and bolus with the required precision rate; verified that occlusion alarms are activated during emergency conditions and the device is safe for patient and operator use. In addition, electrical safety tests were performed.	The quantitative analysis of the variables related to the precision of the flow showed that the results obtained are critical and that the percentage of infusion error can be above 20% depending on the conditions of the equipment.
[28]	A. Cauchi et al., 2013, London	19 BBraun brand infusion pumps and Infusomat Space model.	Record errors in typing prescriptions were analyzed using the 5-key keyboard; the results were used to determine the probability distribution for the errors found.	For the set of infusion pumps studied, a percentage of typing errors of 7.13% was found for the infusion volume (VTBI) and 16.91% for the infusion rate or rhythm (rate).
[29]	S. Taghipour et al., 2011, Canada	Hospital history data from various pumps.	Different failure modes were considered, of different models of infusion pumps, being studied failures of the warning system (audible), chassis and battery.	The authors found very similar results in relation to the reference method, considering censored data.
[30]	K. Giuliano, 2018, United States	Volumetric infusion pump recall data.	Analyzes the number and type of failures in the different classes of infusion pumps.	The researchers point to the need to develop new technologies associated with the design of infusion pumps, aimed at improving usability and safety.
[25]	E. Batista et al., 2021, Portugal	Nexus 3000 infusion pump.	Calibration and uncertainty calculation; validation of the interferometry method, comparing with the gravimetric method.	Determination of uncertainty and validation of the proposed method.
[31]	M. Etelvino et al., 2019, Brazil	371 peristaltic volumetric infusion pumps from two different brands.	They carried out descriptive research using a qualitative approach in a hospital located in Rio de Janeiro. The analyzes were based on IP maintenance records and on collections made from April to June 2017.	As for the record for the last preventive maintenance performed, it was identified that less than 10% of the equipment studied were up to date, 54.5% had an expired preventive maintenance record, 5.9% had an illegible preventive maintenance record and 29.9% lack of preventive maintenance records.
[32]	M. Blancher et al., 2019, France	05 syringe infusion pumps, two of the standard model and three of low weight.	Benchtop comparative study with two different flow measurement methods performed during a 2-h infusion period at altitudes of 300, 1700 and 3000 m.	Lightweight models provide bolus rather than continuous flow. Even though they are 10x heavier, standard devices seem to be more reliable even at different altitudes.
[33]	S. Lee et al., 2018, Korea	Pressure-based pump, syringe pump, and drug delivery device. Syringe pump (Chemyx, Nexus 3000), commercial pump based on pressure (Elveflow).	They evaluated infusion pumps using the dynamic gravimetric method. Flow and pulsating flow stabilities from syringe and infusion pumps were analyzed according to flow rate. The measurement error and its uncertainty were obtained according to the flow.	The authors concluded that pulsating flows and rates can be measured as they interfere with pump operation.
[23]	E. Batista et al., 2019, Portugal	Syringe pumps and peristaltic pumps.	The study investigated the influence of rapidly changing flow rates due to a predefined flow rate change. In addition, a multi-infusion setup is developed to analyze fluid flow rates and their compositions at the exit of the infusion line.	By improving the precision of flow rate measurement of drug delivery devices, with the development of new measurement methods, dosing errors can be reduced. This can be achieved by capturing traceable calibrations of low-flow and ultra-low-flow infusion devices and by better understanding the calibration of dosing administration in clinical settings, especially in the cases of multiple infusion systems.
[34]	S. Manrique Rodríguez et al., 2014, Spain	Smart infusion pumps.	Analysis of failure modes and effects (FMEA) in the pediatric intensive care unit of a General and Teaching Hospital. The FMEA was carried out before the implementation of CareFusion smart infusion pumps and 18 months after identifying the risk points during three different stages of the implementation process: creation of a drug library; using the technology during clinical practice and analyzing the stored data using Guardrails R CQI v4.1 Event Reporter software.	Several improvement actions were carried out, including periodic reviews of the drug library, the development of supporting documents and profile training in the system. Eighteen months after implementation, these measures helped to reduce the likelihood of each risk point occurring and increase the likelihood of its detection.
[24]	E. Batista et al., 2020, Portugal	Syringe pump, brand not identified.	Investigation of different flow regimes, liquid mixing behavior and occlusion phenomena in infusion systems aimed at improve dosing accuracy mainly at flows as low as 100 nL/min.	The results showed that the errors obtained using PP syringes are considerably higher than those obtained with glass syringes, due to the compliance effect. This indicates that PP syringes should not be used in micro flow measurements or in flow generators employed in bio analytical, medical or microfluidic applications unless the entire setup (syringe and flow generator) can be calibrated/evaluated by a recognized laboratory and traceable.
[35]	M. Felipe et al., 2020, Brazil	03 syringe infusion pumps.	The syringe pumps were placed at the distal outlet level of the infusion line, 30 cm above and 30 cm below, to verify how variations in the height and density of the solution can influence the accuracy of the pumps.	The position of the syringe infusion pump can influence the amount of volume infused. The influence was more evident in the low infusion rate. These variants should be considered in intravenous syringe therapy, in infusion pumps in pediatric patients, to reduce medication errors triggered by changes in hydrostatic pressure and in the system.
[36]	M. Baeckert et al., 2020, Switzeland	07 sets of syringe infusion pumps.	The sets were evaluated in an in vitro study during start-up, vertical displacement maneuvers and occlusion of the infusion line at a defined flow rate of 1 ml h$\hat{}$-1. The measured data were used as input to a concentration simulation pharmacokinetic simulation plasma during a continuous neonatal epinephrine infusion pattern.	The problems that affected all tested sets are mainly related to the working principle of the infusion pump syringe and will only be partially resolved by incremental improvements to the existing equipment.
[37]	R. Gandillon 2013, United States	Data from 30000 general purpose infusion pumps from four manufacturers.	The intervention performed was found in a database to analyze the error rate as a function of cost and maintenance time.	By looking at historical repair information and choosing a provider with a low repair rate, many dollars and hours can be saved at a single hospital and, ideally, be reinvested in improving patient care. The study comes as a clinical utility in decisions about medical equipment acquisitions and fostering collaboration between clinical engineers, physicians and equipment manufacturers to improve medical devices, design and improve patient safety.

### Quality and risk of bias individual studies

**Fig. 7:**
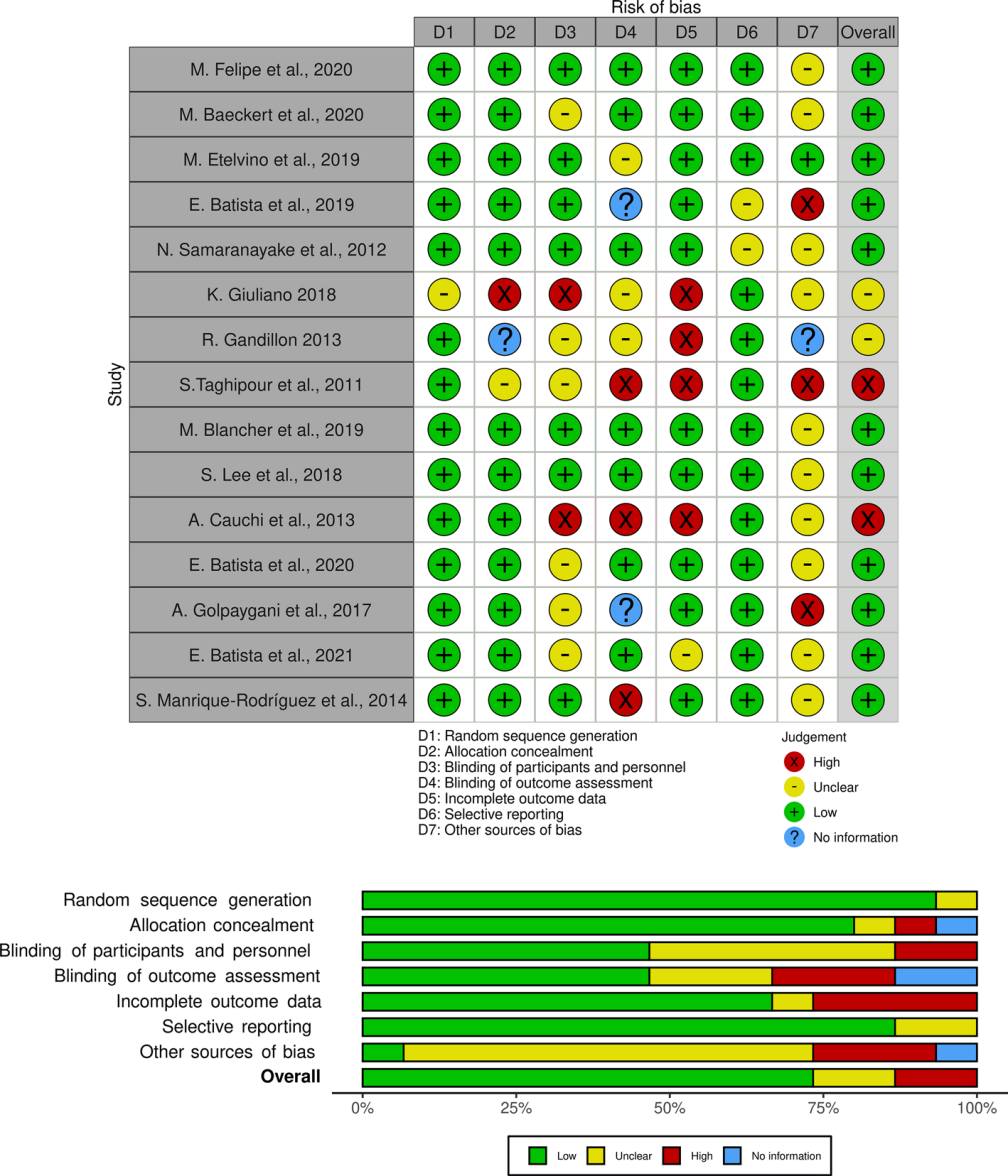
Quality analysis of studies plotted on Robvis using the “Generic” tool. Of the included studies, 11 had a low risk of bias

In evaluating the introduced studies, the Robvis “Generic” tool [22] showed that two studies had a high risk of bias [28, 29]. The domains in which they have problems are associated with 4) blinding of outcome assessment, 5) incomplete outcome data and 7) other sources of bias. We also saw that two other works had some associated problems and were not clear about the overall risk [30, 37], as shown in Fig. 7. In terms of criteria, we used “high” for high risk of bias, “low” for low, “no information” for missing data, and “unclear” when the information provided was incomplete or difficult to understand.

Of the included articles, most present a well-defined study and low risk of bias (*n*= 11). Accordingly, we observed that 14 studies discussed in this systematic review are of high quality for domain 1 and 13 studies for domain 6, both being above 75%, thus adding considerable quality to the included studies.

### Outcomes of the studies

Following quality analysis of the studies, we developed a table that summarizes results of the research outcomes included in this review. In Table 4, we check the studies, the intervention adopted in each one, and their respective outcomes.

**Table 4:** Outcomes of the inserted studies

Study outcomes					
Study	Intervention		Outcomes		
Author, year	Sample	Análise/Ensaio realizado	Precision	Reliability	Others
[26]	1538 incidents with medications reported from 2005 to 2010	The errors related to technology were analyzed to identify the type of technology responsible and the causes. The errors were divided into (i) errors related to technologies; (ii) errors unrelated to technologies. Within the group of errors related to technologies there was the division in: sociotechnical errors (which have human interaction) and device errors (related to technical defects of the device).	Not applicable	Not applicable	This study concludes that during 5 years analyzed most errors were sociotechnical that involved human interaction. The most common causes of tech related errors were: problems at the interface between equipment and user, non-compliance with existing procedures and inadequate procedures.
[32]	Syringe and peristaltic pumps, not informed quantity	The main objective of the study is to allow for traceable volume, flow and pressure measurements of existing drug distribution devices (such as infusion pumps and analyzers) and online sensors that operate at flow rates below 100 nL/min to avoid imprecise measurement results. The study also investigated rapid changing flow rates, liquid mixing behavior and occlusion phenomena in multi-infusion systems to improve dosage accuracy in each infusion line.	The study was developed under a precision linear stage actuator to ensure results in standards themselves.	Not applicable	By improving the measurement precision of the delivery device flow rate of drugs, with the development of new measurement methods, errors of dosage will be reduced and lives will be saved. This can be achieved for the acceptance of a wider spectrum range of traceable calibrations of low flow infusion devices and ultra-low and by advanced knowledge device calibration address of drug delivery in clinical trials, especially in the case of multiple infusion systems.
[34]	Incident reports involving IP of the pediatric intensive care unit of a general hospital and teaching.	The objective of this study was to carry out a FMEA (analysis of failure and effect modes) on the risk points in the use of intelligent IP in a pediatric intensive care unit before and after devices are implemented to identify possible improvement actions and Evaluate the effects that these improvements may have at risk points has detected.	Not applicable	Not applicable	Various periodic revisions of the medicines library, the development of support documents and the inclusion of improvement in the system profile were carried out, so that alarms fired by actual programming errors can be distinguished from those caused by the incorrect use of the system. Eighteen months after implementation, these measures have helped reduce the likelihood of occurrence of each risk point and increase the likelihood of their detection.
[28]	19 IP Brand B Braun Infusomat Space Model	Registration errors were analyzed in typing the requirements, using the 5-key keyboard. The results were employed to determine the distribution of probability for the errors found.	Accuracy is evaluated by the proportion of the occurrence of typing errors	Reliability was not calculated by MTTF times, but can be associated with probability distributions of volume (VTBI) and infusion rate (rate)	The values obtained for reliability provide information for improving the reliability of the equipment via project improvement.
[29]	Historical data from hospital files	The test of the likelihood ratio was used to check trends in system fault data subject to repairs, with censored and uncensored data.	Not applicable	Concludes on the test method of the likelihood ratio, proposed to check trends in faulty data with censorship.	The work proposes to use the likelihood test ratio to verify the trends in faulty data with censorship. A recursive procedure is used to resolve the main technique problem to calculate the expected values in step and requiring numerical integration. The proposed method was used to perform a trend analysis of some components of an audible component general IP, chassis/housing and battery using a hospital maintenance data.
[30]	Infusion pump recalls data	Analyzes the amount and type of failures in the different classes of infusion pumps	Not applicable	Not applicable	The author points to the need to develop new technology associated with the project of infusion pumps, aiming to improve usability and security.
[27]	50 IP of 04 distinct marks, the models were not specified	They performed tests to verify that the device pumps the flow rate, volume and bolus required with the required precision rate. They verified whether occlusion alarms are activated during emergency conditions and the device is safe for use of the patient and the operator. In addition, electric safety tests were made.	Precision of flow rate, accuracy of total infused volume, occlusion pressure, bolus and batteries most of the analyzed pumps had the precision of the flow rate with a percentage error between 10% and 20%, followed by errors above 20%.	Not applicable	They found that they use inadequate infusion sets such as tubes and syringes increases the percentage of error and inaccuracy of 10 to 20%. The position of the droplet detector installed in the inappropriate place increases the percentage of error by up to 25% and low-quality batteries allow the flow rate of 10 to 30%
[24]	Nexus syringe pump	A gravimetric method was used to quantify the measuring error of the water volumetric flow of 1000 $\mu$L/h to 1 $\mu$L/h distributed by a syringe pump in different experimental conditions that replicate the current microfluidic settings.	Connectants of temperature uncertainties, water density, air density, mass density, initial and final time, initial and fine mass, expansion coefficient, evaporation, buoyancy and repeatability were taken from the literature, calibration, certificate or estimation, measures, standards and estimates.	Not applicable	In the article in question, experiments were prepared to test the installation microflux of the Portuguese Quality Institute up to 1 $\mu$L/h and verify that the gravimetric traceability of devices operating in this range can b achieved. In addition, several tests were performed with different types of removable syringes and tubes to evaluate the variation of the measurement error and uncertainty in actual working conditions. The results show that the errors obtained with PP syringes are considerably higher than those obtained with glass syringes due to the adhesion effect.
[25]	Syringe Pump Nexus 3000	It presents comparative data for validation of the method and apparatus developed, interferometry, compared to the gravimetric method. Calibration and calculation of uncertainty; validation of the interferometry method, comparing with gravimetric method	The evaluation of precision is made by measuring uncertainty.	Not applicable	This study can be employed to illustrate the microvider calibration, using interferometry and calculating measuring uncertainty.
[35]	03 syringe infusion pumps	The assay aims to verify how variations in the height and density of the solution may influence the precision of syringe IP. The pumps were studied in two infusion rates of 0.5 ml/h and 10.0 ml/h. The solutions studied were saline and parenteral nutrition. Syringe IPs were placed at the distal output of the infusion line, 30 cm above and 30 cm below.	After 2 h infusion, precision loss was mainly verified at 0.5 ml/h with a significant influence of placement of the infusion pump (p <0.001). 10.0 ml/h, there were differences between the saline and parenteral solution at the same level (p <0.004) and 30 cm above (p <0.001). After 2 h of infusion, the superior the error rate identified was 20.1%.	Not applicable	The article concludes that the position of the syringe infusion pump may influence the amount of infused volume. The influence was more evident at the low infusion rate. These variants should be considered in intravenous therapy with syringe infusion pumps in pediatric patients to reduce medication errors triggered by changes in hydrostatic pressure and system compliance.
[36]	07 Syringe infusion pump sets	Seven series of syringe infusion pump currently marketed were evaluated in an in vitro study during initialization, vertical displacement maneuvers and infusion line occlusion at a defined flow rate of 1 ml H ⌃- 1. The measured data were used As input to a pharmacokinetic simulation simulation of plasma concentration during a continuous neonatal pattern of epinephrine infusion.	The mean time from the beginning of the infusion pump until the steady state flow ranged from 89 to 1622 s. The delivery time of the zero drug after lowering the pump ranged from 145 to 335 s. In all sets tested, occlusion alarm delays and flow irregularities measured during vertical displacement maneuvers resulted in relevant plasma deviations Concentration of epinephrine (> 25%) calculated by the pharmacokinetic simulation model.	Not applicable	The article concludes that problems with the performance of the syringe infusion pump sets may have a considerable impact on the plasma concentration of the drug when highly concentrated short-acting cardiovascular drugs are administered in low streaming quotes. The problems, which affected all. The sets tested are mainly related to the functioning principle of syringe infusion pumps and will be solved only partially by incremental improvements of existing equipment.
[37]	30000 data General use infusion pumps	Analysis of incidence of failures and costs of acquisition and maintenance, lifelong, in 4 types of infusion pumps of a database only from Veteran’s Health Administration (VHA)	Not applicable	The study measures the reliability of the IP of 4 unspecified manufacturers of Veteran’s Health Administration (VHA) database. The study made a caught of the services generated when the maintenance of said pumps was requested.	The author concludes that when observing the percentage of devices with Generic User Error (UES), it is possible to know which manufacturers manufacture pumps with fewer problems people are using. Not only is this low UE occurrence indicate less frustration for nurses’ learning and the use of the new equipment, but this means that the probability for the occurrence of an adverse patient safety event decreases.
[31]	371 Peristaltic Volumetric IPs	The objective of the essay is to carry out a situational diagnosis related to the preventive maintenance of peristaltic volumetric infusion pumps, through a descriptive, quantitative research, carried out in a federal hospital in the city of Rio de Janeiro. 371 peristaltic volumetric IPs, two distinct brands were analyzed.	Not applicable	Not applicable	This situational diagnosis revealed an outdated technological park, with significant lag in relation to the validity of preventive maintenance of peristaltic volumetric IPs. The improvement actions started with the updating of patrimonial data for the amount of peristaltic volumetric infusion pumps from hospital. Finally, monitoring of the conditions of the peristaltic volumetric infusion pump system was initiated, so that there is a continuous control of the conditions of this equipment.
[32]	05 Devices Syringe IP - (two standard and three low weight) to 300, 1700 and 3000 m altitude.	A comparative bench study was performed. The objective of this study was to compare the infusion flow rates provided by low-weight (syringe infusion pumps) versus standard pump devices, in the stage of pre-hospital emergency medicine, at different altitudes.	Interquartile IQR track. Medium infusion flow rates (p = 0.83) and interquartile intervals (p = 0.27) did not differ between syringe infusion pump devices. Median Infusion flow rates (p = 0.15) and interquartile intervals (p = 0.71) did not differ depending on altitude. Medium infusion flow rates (P for interaction = 0.32) and interquantal intervals (P for interaction = 0.66) for syringe infusion pumps did not vary significantly with altitude.	Not applicable	Low weight devices provide bolus instead of continuous flow. Even if they are ten times heavier, standard devices seem to be more reliable even at different altitudes. Only automatic instant flow measurement showed this very important difference between the devices. We believe that there are precision standards may not be relevant to use in the prehospital environment. However, manufacturers must provide instant flow information.
[33]	Pressure-based pump, syringe pump and drug administration device.	In this study, drug delivery devices, namely, IP and syringes, were evaluated by a dynamic gravimetric method. The flow rates of the syringe pump, IP and pressure-based pump were measured in various flow rates (0.5–60 mL / h) using a dynamic gravimetric method and a flow meter.	The uncertainty of the measurement error of each pump was obtained using the gravimetric calibration method tric and flow meters. For the purpose of analyzing the pump pulsating flows of syringe and infusion, the period of pulse and pulse amplitude tion were obtained with respect to the flow.	Not applicable	The article confirms that bombs had distinct performances as a result of their different principles of work. In addition, the dosage rate of the medicine can be altered by various reasons, such as mixed flow, the effect of the blood circulatory flow, and the alvocontrolled infusion method. Therefore, these pulse flow rates can be measured using the dynamic gravimetric method in medical applications

## Discussion

### Data and infusion pumps analyzed

This was a systematic review of 15 studies that evaluated the condition of infusion pumps. The review investigated whether accuracy and reliability had been analyzed during the last 10 years. We verified that to proceed with these types of analysis, it is not always necessary to have the equipment or to know specifically the manufacturer/model. In the studies [26, 29, 30, 34 and 37] the data considered referred to reports, databases, hospital files, recall data, and other files capable of supporting information for the evaluations. Based on this information, the studies followed different lines to investigate operation of the pumps.

Of the included studies, 10 had specific IPs to evaluate and explore in general. Study [23] tested syringe and peristaltic pumps to investigate different flow methods, occlusion phenomena, and other parameters, to improve infusion accuracy. Syringe IPs are used with disposable syringes and have a complex system of hardware and software designed to monitor the infusion process, probability of infused liquid errors, pump information and other parameters [38]. In this pump model, the syringe acts as a reservoir for the fluid and most of the time its capacity is 60 ml or less. The syringe plunger moves from the pump controls that push fluid and regulate its flow [3].

Studies [24, 25, 32, 33, 35 and 36] also considered different aspects of syringe pumps. Study [24] applied a gravimetric method to assess flow rate of syringe pumps under different conditions and in their study the following year used interferometry to compare with the gravimetric method [25]. Another study that addressed syringe BI associated with the dynamic gravimetric method was proposed by [33]. In it, the pumps were evaluated and error and uncertainty were obtained through the flow. It was concluded that pulsating flows and rates must be measured as they interfere with pump operation. The technique of evaluating BI using gravimetry was identified in 3 studies.

In study [35] infusion accuracy was also investigated for infusion accuracy. However, they were subjected to tests in different environments with variations in height and density of the substance, to determine if such factors would influence precision. For this purpose, pumps were placed at the distal infusion outlet level and later moved up and down. In addition, using syringe pumps, study [36] performed vertical displacement maneuvers and occlusion of the infusion line, to conclude that the problems that affected the tested sets can be partially solved with incremental improvements to the equipment. As well as in studies [35 and 36]. In study [32] the impact of height variation on the flow rate during the use of syringe pumps is also evaluated, reaching similar conclusions. Thus, we can see that the positioning of the equipment can interfere with its operation and has been considered in the literature.

In addition to the use of syringe pumps, we identified error analysis adopting peristaltic BI. The peristalsis propulsion model is the most commonly used, as it allows for a constant flow of substances without damage to liquids and its flow will depend on the thickness of connected tubes. The main limitation of peristaltic pumps is the high cost of the equipment [39]. Of the listed studies [28 and 31] evaluated pumps of this category. The analysis of [28] discusses data entry errors in digital displays. The data refer to registration errors when typing prescriptions using the 5-key keyboard and the results were used to determine probability distribution for the errors found. Study [31] also used a peristaltic pump to carry out a situational diagnosis with more than 300 pieces of equipment. This approach focused on preventive maintenance through descriptive and quantitative research. The diagnosis revealed a lag and outdated preventive maintenance for a technology park at a federal hospital in Rio de Janeiro, Brazil. Such findings present evidence of a lack of precision and reliability in the infusions performed in the environment, where the study was carried out and further reinforce the need for periodic maintenance.

Despite adopting a quantitative measure of 50 IPs, study [27] does not identify the characteristics of the evaluated equipment. In this study, tests were performed to examine if the flow rate and volume are compatible with the schedule and if the alarms are working properly. In addition, they consider fundamental aspects of being assessed, which relates to the guarantee of patient and operator safety. It is noted that the equipment model is a factor to be considered in studies aimed at precision and reliability, as well as the position of use.

### Analysis of precision and reliability of studies

Medical products must go through the following phases to achieve reliability: analysis, testing, associated areas, reliability measures, and failure data [40]. Fogliatto et al. details that a reliability program aggregates the institution of procedures and routines to manage dependability in four phases of a product’s life: design and development, manufacture and installation, operation and maintenance, and disposal [41]. Thus, in this study, we specifically evaluated the operation and maintenance phase.

Among the selected studies, [26, 30, 31 and 34 do not have direct interventions for accuracy or reliability analyses. However, some variables are correlated with safety of infusions. The study [26] evaluates pump-related errors and divides them into (a) technology-related errors and (b) non-technology-related errors. In (a) there was a division between sociotechnical errors (which have human interaction) and device errors (related to the technical defects of the device). Equipment errors and failures must be extensively investigated and discussed, as they are obstacles to achieving accuracy and reliability. Human errors can be minimized with qualified labor and training, while technical errors require high rigor in the product design and development stage.

In study [34] incident reports involving IP were evaluated using Failure Modes and Effects Analysis (FMEA) techniques. This technique made it possible to detect risk points in the use of pumps and the variables associated with failures, which are essential in the investigation and comprise the methods for measuring reliability [41]. On the other hand, considerations in study [30] were made from IP recalls, which examined the number and type of failures in various pumps. The study also addresses factors of administration errors and usability, arguing that innovation and technology development should be associated using the human factors approach.

Finally, study [31] did a situational diagnostic search related to preventive maintenance of the pumps. Through descriptive and quantitative research, they found that less than 10% of the pumps’ technological parks were up to date in terms of maintenance, 54.5% had expired preventive maintenance records and 29.9% had no record of maintenance at all. The results and conclusions of study [31] illustrate negligence with equipment that can cause damage to health, in a basic principle that is preventive maintenance. Therefore, the reliability and accuracy of the equipment is not guaranteed for either the operator or the patient.

Study [42] notes that precision can be defined as the degree of conformity between a series of observations of the same random variable, and that the spread of the probability distribution is an indicator of precision. In this context, study [23], analyzing syringe infusion pumps, investigated rapidly changing flow rates, liquid mixing behavior, and occlusion phenomena in multi-infusion systems, aiming to improve the accuracy of delivered dosage, particularly for flows as low as 100 nL/min. The study concludes that by improving the accuracy of flow rate measurement in drug delivery devices, with the development of new measurement methods, dosing errors can be reduced. This can be achieved by a broad uptake of traceable infusion calibrations and enhanced knowledge of calibration for drug delivery devices in clinical settings, especially in the case of multiple infusion systems.

Similarly as in the previous one, also focused on flow, study [27] performed tests to verify flow rate, volume, and bolus delivered by the device at a required accuracy rate. They verified the safety of the device for the patient and operator, as well as confirming that occlusion alarms are activated during emergency conditions. The study also investigated the electrical safety of infusion pumps.

Another study that approaches flow as a theme is number [24]s. Study [24] investigates the influence of rapidly changing flow rates in general with the gravimetric method, due to a predefined flow rate change. In addition, a multi-infusion setup is developed to analyze flow rates of fluids and their compositions at the exit of the infusion line. They concluded that dosing errors can be reduced by improving flow rate measurement of devices and that this can be achieved by capturing traceable low-flow and ultra-low-flow infusion calibrations, in addition to a better understanding of dosage administration calibration in clinical environments. As in the previous study, [33] adopted the gravimetric method to analyze the IP. The authors conclude that pulsating flows and rates can be measured as they interfere with pump operation. They confirmed that the pumps performed differently as a result of their different working principles. These results can be used for medical and biological applications.

In 2021, study [25] brought new innovations to the evaluation process of the equipment. They performed calibration, the uncertainty calculation, the validation of the interferometry method, and compared it to the gravimetric method. From the obtained results, the authors concluded that the gravimetric method can be used for calibration of flow devices up to 0.01 mL/h with a reasonable uncertainty value of 5% (*k* = 2). They reported that the interferometer method appears promising in allowing for reliable, reproducible, and more certain measurements in the microliter flow domain. In this way, future studies may make use of better measurement of syringe radius, executing including measurements of up to 0.1 $\mu$L/h, as well as testing other types of flow generators, and validating the interferometer methodology by a comparison.

In studies [35, 36], and [32] research was successful using syringe pumps. In the first one, 03 pumps were placed at the distal outlet level of the infusion line, 30 cm above and 30 cm below, to verify how variations in the height and density of the solution can influence the accuracy of the pumps. The study concludes that the position of the syringe infusion pump can influence the amount of volume infused, especially at low infusion rates. The second study worked with 07 syringe infusion pump sets, which were evaluated in an in vitro study during start-up, displacement maneuvers, and occlusion of the infusion line at pre-defined flow rates. The problems that affected all tested sets are mainly related to the working principle of the syringe of the infusion pumps and will only be partially solved through incremental improvements to the existing equipment.

The search in study [32] tested 5 syringe infusion pumps and 3 lightweight pumps. They performed a bench top comparative study with two different flow measurement methods, performed over a 2-h infusion period between amplitudes of 300 and 3000 ms. They noted that low-weight syringe infusions provide discontinuous flow with potential clinical implications for critically ill patients receiving vasoactive drugs. This study also highlights a hitherto unknown negative impact of altitude on pump function. For these reasons and problems related to syringe IBs, these variants should be considered in intravenous therapy especially for pediatric patients, to reduce medication errors triggered by changes in hydrostatic pressure and the compliance system.

In terms of equipment reliability, study [28] analyzed registration errors in typing prescriptions, using a 5-key keyboard, where results were used to determine probability distribution of the respective errors found. A percentage of typing errors of 7.13% was found for infusion volume and 16.91% for the infusion rate. These results led the authors to conclude that for the set of infusion pumps studied, by combining real empirical data with a new approach for studying usage error, this article raised several questions about medical device design, as well as lamenting the scarcity of data available to increase design priorities reducing error magnitudes and error rates.

The search of study [37] corroborates results of study [28] on the importance of having databases with information on medical equipment. The author states that, with this information, it is possible to investigate reliability and usability of the instruments reported. Because of this she carried out a study to investigate the effect of the infusion pump models on operating errors, based on stored historical data, considering pumps from four different manufacturers. To compare the equipment, she determined the failure rate and operating errors (usability measure) of each model. From the number of devices and installations, graphical analysis, by histograms, was performed to evaluate errors and usability, corrective maintenance and reliability, in addition to cost and time savings. In regards to records of infusion pumps that had been in operation for at least 1 year, and had undergone at least one corrective maintenance procedure were considered for analysis. The graph shows significant differences between manufacturers and increases over the life of the equipment. As for errors and usability, only one device showed a significant difference in relation to the others. In conclusion, the usability and reliability measures can be considered as very useful information for the acquisition of new equipment by hospitals and also for the improvement of the project by the manufacturer.

Following the area of reliability in infusion pumps, the study authors [29] verified changes in the performance of different repairable components present in the equipment. Two different types of failures were considered: those that interrupt the instrument’s operation, such as failures in indicators and relays and the occlusion of fluid flow channels and those that alert you to a fault but do not interrupt operation, such as defects in electronic circuits and audible operating signals. In this research, historical data on failures of components of infusion pumps were used, taking into account variables, such as sound signal, fairing, and battery. Graphical methods and trend tests were used for analysis. The authors review the trend tests presented in the literature and proposed a statistical test to verify the null hypothesis that the failure process follows a non-homogeneous Poisson process (PNHP). Up to this point these methods had only previously been studied with rightcensored data, and their use was proposed assuming a powerlaw density function, with the expectation–maximization (EM) algorithm applied to estimate the model parameters and compared with the modified MS. Three case studies were carried out for the mentioned components, and 80 random data sets were used for the component that beeps, 38 for the fairing, and 674 for the battery. The results showed that the parameters estimated by the EM and modified EM methods were very close.

## Limitations

In preparing this systematic review, some limitations were noted. First, there was heterogeneity in the analysis of failures, characteristics of error records, models, maintenance period, and parameters evaluated which made the meta-analysis impossible. Most studies did not clearly specify the methodology, tools, and calibration of maintenance instruments, which are necessary for risk of bias and quality, assessment.

## Conclusion

This review intended to investigate whether precision and reliability are aspects that have been evaluated in recent years. The research reviewed here points to the need for periodic monitoring of flow rate of the equipment, especially for low flows. This variable is essential to maintain patient safety and reduce dosage errors. In addition, it is noted that the gravimetric method is a proposal that delivers positive results in the evaluation of the flow, being an alternative for those responsible for clinical hospital engineering to utilize in routine maintenance of the devices.

Based on the findings of this review, it is noted that there is continuous need for new developments in studies that clearly adopt concepts related to reliability. In possession of the results, we verified the lack of the area applied to infusion pumps, principally studies that involve bench tests and in loco. This deficit promotes inaccurate infusions, an increase in adverse effects, and possible harm to the patient.

## Supplementary Information

**Additional file 1.** Exclused items and rasons for exclusion.

## Data Availability

The data sets used and/or analyzed in the current study are available upon request from the corresponding author. The original contributions presented in the study are included in the article/Supplementary Material. Further inquiries can be directed to the corresponding author.

## Abbreviations

IP: Infusion pump
IEEE: Institute of electrical and electronic engineers
ICU: Intensive Care Units
NICU: Neonatal Intensive Care Units
