# Supplementary material for: Precision and reliability study of hospital infusion pumps: a systematic review
Source: Biomed Eng Online. 2023 Mar 17;22:26. doi: 10.1186/s12938-023-01088-w (PMC10023007; doi:10.1186/s12938-023-01088-w)
Supplement: Supplementary file 1 — Additional file 1. Exclused items and rasons for exclusion. [file 12938_2023_1088_MOESM1_ESM.pdf]

Additional file 1 - Exclused items and rasons for exclusion

**Reason for Exclusion**	**Quantity**
1. Did not meet the outcomes of the PICO acronym	48
2. Implantable infusion pumps	12
3. Other languages that do not meet the inclusion criteria	7
**Total**	67
